# Does hypothalamic SIRT1 regulate aging?

**DOI:** 10.18632/aging.100311

**Published:** 2011-03-18

**Authors:** Giorgio Ramadori, Roberto Coppari

**Affiliations:** ^1^ Department of Internal Medicine, Division of Hypothalamic Research, The University of Texas Southwestern Medical Center, Dallas, TX, 75390, USA; ^2^ Faculty of Medicine, Universita’ Politecnica delle Marche, Ancona 60020, Italy

**Keywords:** hypothalamus, SIRT1, aging, metabolism, brown adipose tissue, obesity

## Abstract

In virtually all organisms, life expectancy is profoundly affected by caloric intake. For example, dietary restriction (DR; a feeding regimen of fewer calories compared to the ad libitum level without causing malnutrition) has been shown to lengthen, whereas hypercaloric (HC) diet feeding to shorten, lifespan. Recent findings in invertebrates indicate that specialized groups of cells (e.g.: metabolic-sensing neurons) detect changes in caloric intake and convey energy-status-variation signals to other cells in the body to regulate lifespan. In mammals, whether metabolic-sensing neurons govern aging in a cell-non-autonomous fashion is unknown. Yet, this is a captivating and testable hypothesis.

There is little doubt that caloric intake impacts on aging. DR has been shown to prolong lifespan in a myriad of organisms including yeast [1], worms [2], flies [3], fishes [4], and rodents [5]. Also, in monkeys DR has been reported to reduce the incidence of metabolic defects (e.g.: insulin resistance and augmented adiposity), cancer, cardiovascular disease and brain atrophy all of which are established age-related illnesses [6]. Despite this study has yet to be completed it has provided compelling evidence supporting the notion that DR lengthens lifespan in primates and eventually in humans as well. On the contrary, HC diet feeding leads to metabolic imbalance, accelerates the pace of aging, and hence shortens lifespan [7]. Unfortunately, a significant fraction of the human population frequently feeds on calorie-rich foods. This practice has critically contributed to the obesity and type II diabetes mellitus epidemic seen in recent years [8] and is expected to reverse the remarkable trend of increased life expectancy recorded since the beginning of the last century. Therefore, developing effective strategies aimed at improving quality and duration of life in current calorie-rich conditions will require a better understanding of the mechanisms (cells and molecules) governing aging and protecting from diet-induced metabolic imbalance. Toward these ends, the cell-type(s) and molecule(s) underlying the effects on metabolism and lifespan exerted by low or high caloric intake must be unraveled. Below, we will discuss the possibility that specialized metabolic-sensing neurons in the hypothalamus are key players of these pathways. The roles of SIRT1 in hypothalamic neurons on metabolism and aging will also be touched upon.

Recent results have bolstered the idea that cell-non-autonomous mechanisms link changes in energy intake to lifespan. Bishop and Guarente have provided experimental evidences that neuroendocrine cells are required to link DR to longevity in the worm *Caenorhabditis elegans* [9]. Also, Durieux and colleagues have shown that manipulation of the electron transport chain only in neurons modifies lifespan of the worm [10]. In the fly *Drosophila melanogaster*, overexpression of either dSir2 (an ortholog of mouse SIRT1, see below) or uncoupling protein 2 (a protein that uncouples oxidative phosphorylation from ATP production and is key for normal glucose-sensing in mammalian neurons [11]) only in neurons leads to increased lifespan [12, 13]. In mammals, the hypothalamus contains neurons able to detect variations in body's energy status and relay this information to downstream neuronal and non-neuronal cells with the goal of maintaining normal energy, glucose, and lipid balance (hereafter we will refer to this balance as to metabolic homeostasis). Some of these neuronal populations have been biochemically categorized. For example, hypothalamic arcuate nucleus neurons known to suppress or increase body weight include pro-opiomelanocortin (POMC)- and agouti-related peptide (AgRP)-expressing cells, respectively [8]. Because metabolic-sensing mechanisms (e.g.: leptin-, glucose-, insulin-sensing mechanisms) in these neurons have been shown to affect glucose metabolism in peripheral organs, POMC and AgRP neurons are also considered to be critical regulator of glucose homeostasis [8]. Interestingly, defects in metabolic-sensing mechanisms in POMC and/or AgRP neurons lead to increased body adiposity and insulin resistance that (as mentioned above) are typical age-related disorders. It is therefore tantalizing to suggest that the integrity of metabolic-sensing in hypothalamic neurons is crucial for maintaining metabolic homeostasis and hence normal organismal aging.

At the molecular level, members of the Sirtuins family have been proposed to be essential regulators of lifespan [14]. Sirtuins are nicotinamide adenine dinucleotide (NAD^+^)-dependent enzymes known to regulate gene expression and cellular function [14, 15]. The founding member of this family is the yeast protein deacetylase silent information regulator 2 (Sir2) [16]. There are seven mammalian Sir2 orthologs (SIRT1-7) [14, 15]. Due to their dependence to NAD^+^and effects on the transcriptome, Sirtuins are thought to be molecular links between DR and longevity [14, 15]. Bolstering this notion are some experimental evidences. For example, genetic manipulations of Sir2, and its ortholog in fly, influence DR-induced longevity [17, 18]. As mentioned above, overexpression of dSir2 only in neurons extends lifespan in *D. melanogaster* [12]. Also, in mice the DR-induced increase in ambulatory movements requires SIRT1 [19]. Altogether, these data suggest that neuronal Sirtuins may be important regulators of the organismal aging process.

SIRT1 is abundantly and specifically expressed in metabolic-sensing neurons, including hypothalamic POMC and AgRP neurons [20, 21]. To directly test the functional relevance of SIRT1 in metabolic-sensing neurons on aging, mice bearing neuron-type-specific loss- or gain-of-function SIRT1 mutation will need to be generated. The effects of deletion of SIRT1 from either POMC or AgRP neurons on metabolic homeostasis have been reported; however, whether these mutations affect longevity is yet to be known [21, 22]. While we wait for these results to be available some guesswork can be made. For example, POMC-neuron-specific SIRT1 knockout mice display hypersensitivity to diet-induced obesity; a defect brought on by reduced sympathetic nerve activity and brown adipocytes content selectively in one visceral fat depot: the perigonadal fat [22]. It is important to emphasize that brown adipocytes are different from white adipocytes morphologically (brown cells contain multiple lipid droplets whereas white adipocytes have a sole large lipid droplet) and functionally (brown cells are highly oxidative as they convert chemical energy into heat *via* uncoupled respiration whereas white adipocytes stockpile energy) [23]. Interestingly, the amount/activity of brown adipose tissue (BAT) greatly varies with age: BAT content/activity is high early-on in life while it tends to decrease by age [24, 25]. Of note, we have also found an age-dependent decline in BAT in perigonadal fat; an effect that is however not accelerated by HC diet feeding (Figure 1). Our data suggest that in the context of HC diet protective mechanisms against visceral BAT loss are in place and that SIRT1 in POMC neurons is a critical component of these pathways [22].

**Figure 1.**
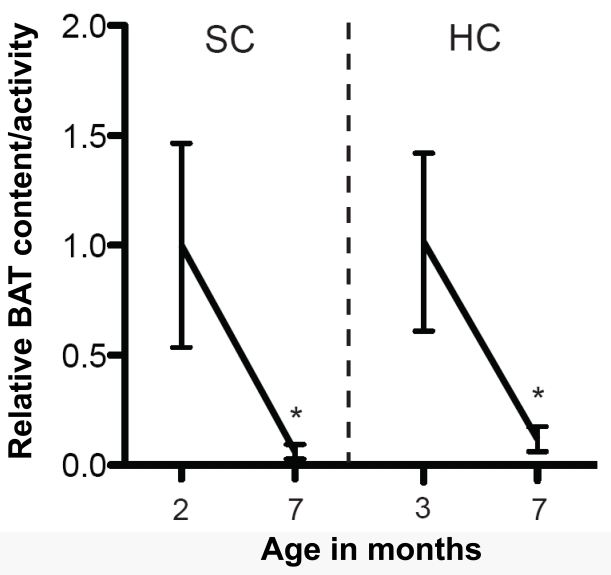
Age-dependent decline in BAT content/ activity in perigonadal fat depot The mRNA level of the brown adipose tissue (BAT)-specific gene uncoupling protein 1 (*Ucp1*) was used as a readout of BAT content/activity in perigonadal fat. *Ucp1* mRNA levels in perigonadal fat of 2- and 7-month-old *Sirt1*^loxP/loxP^ females fed on a standard chow (SC) diet and 3- and 7- month-old *Sirt1*^loxP/loxP^ females fed on a high calorie (HC) diet [22] (n=9 in each group). Individual mRNA levels were normalized to *36B4* mRNA contents. HC-fed mice were fed on a SC diet up to 2 months of age and then switched and maintained on a HC diet. Note that perigonadal BAT content/activity similarly declines with age in SC and HC diet feeding conditions. Statistical analyses were done using two-tailed unpaired Student's t test. * P <0.05.

Due to the established roles of BAT on energy balance, the temporal decline in BAT content/activity could in principle contribute to the age-dependent increased propensity to accumulate adiposity. Because SIRT1 in POMC neurons selectively coordinates BAT in perigonadal fat SIRT1 in these neurons is well-placed to regulate at least one aspect of the aging process (i.e.: BAT decline in visceral fat). Seen from an “aging research” prospective, young-adult mice lacking SIRT1 in POMC neurons display an aged-like perigonadal fat because BAT in this tissue is virtually absent [22]. As mentioned above, this defect contributes to the development of energy imbalance (that is also a typical age-related illness) in the context of HC diet feeding. Thus, POMC neurons seem to regulate crucial aspects of the aging process in a peripheral tissue (BAT decline in perigonadal fat) and of the entire body (increased body adiposity). Whether the metabolically defective perigonadal fat tissue would have an impact on the lifespan of the organism is still unknown. However, due to their increased adiposity mice lacking SIRT1 in POMC neurons are expected to have a shorter lifespan compared to normal controls, at least in the HC diet feeding context that is the peculiar dietary environment of industrialized societies. If this prediction is true, in addition to be a key molecular component of defensive mechanisms against diet-induced obesity SIRT1 in POMC neurons will also need to be considered as a critical regulator of lifespan. Of note, hypothalamic neurons control other age-dependent parameters as for example hepatic and skeletal muscle insulin sensitivity that are also known to deteriorate with age. Indeed, *via* finely tuning vagus nerve activity hypothalamic neurons (including AgRP neurons) have been shown to orchestrate hepatic insulin sensitivity [26, 27]. Moreover, ventromedial hypothalamic (VMH) neurons have been reported to convey metabolic signals to skeletal muscle and as such coordinate insulin sensitivity in this tissue [28]. SIRT1 is expressed in AgRP and VMH neurons [20, 21], yet the relevance of SIRT1 in these cells on hepatic and skeletal muscle insulin sensitivity is unknown. Experiments aimed at directly testing the role of SIRT1 in these hypothalamic neurons on peripheral insulin sensitivity are needed. In addition to address an important question in the metabolic research field, results from these studies will eventually provide the experimental rationale for pursuing (or not) long-term studies designed to testing if hypothalamic SIRT1 also regulates aging. While we wait for these experiments to be completed the hypothesis that the metabolic-sensor protein SIRT1 in metabolic-sensing hypothalamic neurons is a key component of cell-non-autonomous mechanisms underlying lifespan remains an intriguing possibility (Figure 2).

**Figure 2.**
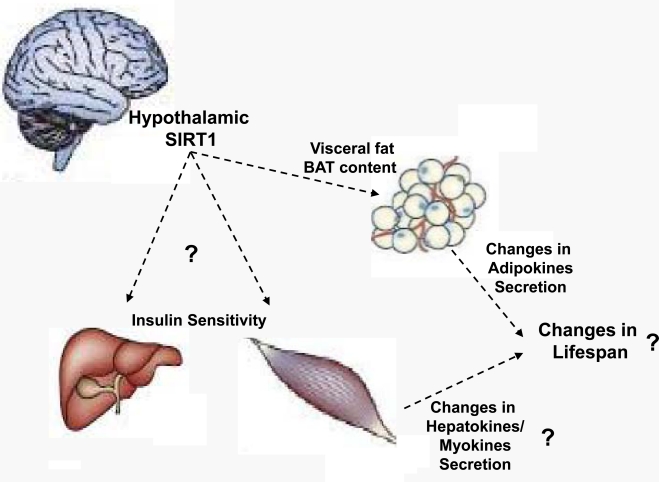
Proposed model by which hypothalamic SIRT1 may control lifespan Hypothalamic neurons control insulin sensitivity in liver and skeletal muscle and the content/activity of brown adipose tissue (BAT) in visceral fat. Recently, we have shown that SIRT1 in hypothalamic POMC neurons governs BAT in perigonadal fat. The model depicted herein predicts that hypothalamic SIRT1 regulates aspects of the metabolic aging process in peripheral organs (e.g.: insulin sensitivity in liver and skeletal muscle and BAT content/activity in fat depots) and by doing so influences the amounts of circulating adipo-, hepato-, and myo-kines which ultimately contribute to changes in organismal lifespan. Experiments aimed at directly testing whether SIRT1 in hypothalamic neurons controls hepatic and/or skeletal muscle insulin sensitivity, and/or exerts any effects on longevity are warranted.
